# Alkali-silica Reaction Elimination Potential of High-Performance Concrete Containing Glass Powder

**DOI:** 10.3390/ma15196574

**Published:** 2022-09-22

**Authors:** Diana Mariaková, Klára Anna Mocová, Kristina Fořtová, Tereza Pavlů, Petr Hájek

**Affiliations:** 1 University Centre for Energy Efficient Building, Czech Technical University in Prague, 273 43 Bustehrad, Czech Republic; 2 Department of Environmental Chemistry, Faculty of Environmental Technology, University of Chemistry and Technology Prague, Technicka 5, 166 28 Prague, Czech Republic

**Keywords:** alkali-silica reaction, high-performance concrete, glass powder, recycling

## Abstract

This study is mainly concerned with the assumption that glass powder can eliminate the potential alkali-silica reaction in high performance concrete. Glass is often land filled, produced as a secondary raw material or as a by-product of production. Chemical analyses were carried out, and the ecotoxicity of the material was investigated, serving as a basis for testing a potential alkali-silica reaction. High performance concrete (HPC) containing different types of waste powder (secondary raw material from production (SGP), jewelry production (SGJ), container waste glass (CWG), and glass from used photovoltaic panels (GPP)) are tested according to the international standard ASTM C1260 and the Czech technical condition TP 137. Newly designed mixtures are innocuous from the ASR point of view in the most cases, except SGP HPC.

## 1. Introduction

Terms such as environment, sustainability, and circular economy appear frequently in scientific works due to the trend towards reducing air pollution, reducing the impact of industry and production on nature, and towards recycling and the reuse of raw materials.

This research deals with the threat of the alkali-silica reaction (ASR) in high-performance concrete (HPC) containing glass powder (GP) and directly follows on from the previous pilot research by the authors about the ASR [1] and a detailed study of the properties of HPC containing GP [2,3,4,5]. The authors are actively dealing with recycled aggregates, secondary raw materials, and waste materials during this extensive research in order to acquire sufficient knowledge in all interdisciplinary contexts [6,7]. Since the beginning of the research in 2017, the attention and volume of data that are available on this issue has increased considerably and it is suitable to compare data among individual countries. Due to the effort to use local raw materials, the results may differ slightly between individual countries.

Concrete is still the number one used in the construction industry and is closely linked to negative environmental impacts [8]. It is mainly caused by the production of cement-based materials. However, modern procedures and approaches are implemented to reduce high energy production and CO_2_ emissions which are closely linked with concrete production [9,10,11]. The popularity of concrete is still increasing and the ways to eliminate its negative environmental impact are examined—among the widely tested methods on the way to green concrete is the replacement of different contents of the concrete composition (for example, cement or fine/coarse aggregate) with other materials with similar properties (mechanical, chemical, and physical), resulting in the design of completely new mixtures.

Glass is tested as one of the highly significant materials in the Czech Republic with a very rich history and is widely used in industry and the construction sector. Glass powder is produced as a waste material (WM) and as a secondary raw material (SRM) during production. These materials often have no further use because of insufficient cleanliness, undesirable color, or other. However, glass is natural pozzolan and does not decompose because it is non-degradable in nature [12]. The use of glass powder in concrete can thus hypothetically contribute to reducing the need for landfill sites, saving money and energy resources. Nevertheless, glass ranks among alternative additives to cement, such as supplementary cementitious materials [13]. WM or SRM utilization in concrete increases the need for thorough chemical analysis and overall toxic safety, especially in relation to nature. Ecotoxicological analysis elucidates interactions in biological systems and shows whether alternative concrete mixtures with glass substitutes can reduce the potential toxic effects of glass itself, as both glass and concrete are associated with toxicity that is related to heavy metal leaching or higher pH [2]. These facts often lead to problems with corrosion, dermatitis, or other reactions such as ASR [14]. 

Ongoing research deals with the ASR mechanism, the conditions affecting this reaction, and the methods that are used for testing. As one of the main degradation processes, ASR damages harden concrete and occurs in concrete under certain conditions. The first identification and initial description of this process was done by Stanton in 1930–1940 [15]. Long-term observations brought new attainment—the alkaline environment and the presence of the reactive aggregate in concrete are the initiators of an alkali-silica reaction. By using Portland cement, an alkaline environment is created due to the presence of Ca(OH)_2_ and although Ca(OH)_2_ represents about a quarter of all hydration products, it can be said that the alkali content (specifically Na_2_O and K_2_O) is much more important in the ASR issue [16]. The problem arises when the reactive aggregate in the resulting strongly alkaline environment begins to form a gel, which creates tension in the concrete and often leads to the formation of cracks.

The chemistry of ASR is a complicated process and was clearly summarized in 2005 by Chatterji et al. [16]. The effort was to understand the mechanism through simple chemistry and to focus on a simple chemical test method to verify ASR. Factors affecting ASR were extended from humidity, temperature, time, and material to the factors such as diffusivity, source, and concentration of the relevant ions. 

The measurement and evaluation of the alkali-silica reaction are usually performed according to standards. The American standard [17], the British standard [18], or the Czech standard [19] sets the conditions for laboratory testing—various boundary conditions are given and the main point of interest is the change in the length of the samples, from which the expansion rate is then numerically evaluated. There are, therefore, studies that deal with ASR within these standards and thus evaluate the impact according to the given regulations. These available studies deal mainly with bottles’ waste glass (container waste glass) as a concrete content, and researches are describing the role of container glass in controlling ASR in concrete [20,21,22,23]. A representative of these type of studies is Dhir et al. [20], who investigated the alkali-silica reaction using the British Standard BS 812-123 [18] with the aim to outcome with specifications for container waste glass (CWG) in concrete. CWG was used as a fine aggregate or filler replacement in concrete. When green and amber glass was used as a fine aggregate replacement, significant expansion occurred. 

The potential alkali-silica reaction is necessary to investigate because it is a long-term process—the damages appear years after the construction [24]. There is an assumption that glass could eliminate the process of ASR. However, the question is whether a high concentration of NaOH masks the released alkali from glass [20]. Another assumption is that particles that are smaller than 1 mm have lower reactivity; the pressure is so low that no cracks appear on the surface [24].

The effort of this research is to summarize the findings of short-term tests according to the American standard ASTM C1260 [17] compared to the Czech technical condition TP 137 [25].

## 2. Materials and Methods

### 2.1. Materials

The tested samples were GP and reference silica flour (SF), which were prepared in the laboratory (each 100 g). A total of four types of glass powder are used in this project and were selected based on the need to recycle, reuse, or use them up:

- container waste glass = CWG,
- secondary raw material from glass jewelry production = SGJ,
- secondary raw material from glass production = SGP,
- glass from used photovoltaic panels = GPP.

The finest fraction of glass is used as a SF replacement to create high-performance concrete and verify the chemical properties, ASR expansion, and structures regarding the mechanical and durability properties from previous research. The replacement ratio was 50% and 100%. Therefore, 9 mixtures were made and tested:

- reference high-performance concrete mixture = REF HPC,
- high-performance concrete containing 50% replacement of silica flour with container waste glass = CWG HPC 50,
- high-performance concrete containing 100% replacement of silica flour with container waste glass = CWG HPC 100,
- high-performance concrete containing 50% replacement of silica flour with secondary raw material from glass jewelry production = SGJ HPC 50,
- high-performance concrete containing 100% replacement of silica flour with secondary raw material from glass jewelry production = SGJ HPC 100,
- high-performance concrete containing 50% replacement of silica flour with secondary raw material from glass production = SGP HPC 50,
- high-performance concrete containing 100% replacement of silica flour with secondary raw material from glass production = SGP HPC 100,
- high-performance concrete containing 50% replacement of silica flour with glass from used photovoltaic panels = GPP HPC 50,
- high-performance concrete containing 100% replacement of silica flour with glass from used photovoltaic panels = GPP HPC 100,

The concrete mixes were designed on the basis of previous research on the basic properties, particle size distribution, and evaluated results. The mixes that were used in this research are summarized in Table 1. Samples of size 25 mm × 25 mm × 285 mm were made according to the standards, always in a set of three pieces. For testing the chemical and ecotoxicological properties, 27 cubes (3 cubes of each mixture with the dimensions 50 mm × 50 mm × 50 mm) were prepared. Accompanying samples were made to verify the constancy of mechanical properties compared to early research [26].

### 2.2. Methodology

The chemical composition of GP was performed by the X-ray fluorescence method using XRF spectrometer ARL 9400 (ThermoFisher Scientific, Waltham, MA, USA). Leachates of both GP and HPC were prepared according to [27]. The chemical and ecotoxicological characterization procedure of leachates is described in [2]. 

There were two experimental methods that were chosen in order to objectively compare the process and the results of the alkali-silica reaction. One of them is the international American standard [17] and the second is the Czech technical condition by the Ministry of Transport [25]. Both are described below.

#### 2.2.1. ASTM Method C1260 

This internationally used method is based on the NBRI accelerated method [17]. The evaluation is possible after 16 days; nevertheless, after this time, it is advisable to continue the measurements for a few more days to improve the reliability of the results. The dimensions of the samples are 25 mm × 25 mm × 285 mm, which is a very subtle element that is under a lot of loads in the extreme environment of this test. The samples are removed from the molds after 24 h and the initial reading is taken. After the initial reading, the samples are placed in the water for 24 h. The samples have to be fully immersed, not touching each other, and be completely surrounded by 80 °C water. After 24 h, the samples are taken out of the bath and the surface is dried. A zero reading is done and the specimens are placed in 1N NaOH (PENTA s.r.o., Prague, Czech Republic) solution. The conditions are similar to those before—the samples have to be fully immersed, completely surrounded by 80 °C NaOH. After 24 h the first measurement is made and another three intermediate measurements are taken between the first and the last, which is after 14 days. If the value is not stable after 14 days, it is advisable to continue with the measurements once a week until the stabilization or disintegration that is caused by massive cracks. 

#### 2.2.2. TP 137 

This Czech technical condition, published in 2016 by the Ministry of Transport, describes the ASR testing [25], which is processed according to ASTM C1260-14 and Alkali-Richtlinie (Deutscher Ausshucss fur Stahlbeton-Richtlinie Vorbeugende Massnahmen gegen schadigende Alkalireaktion im Beton, April 2010) [28]. This method is similar to ASTM C1260 that is mentioned above and also is used in our laboratory. The experiment conditions are the same. The specimens of specific dimension (25 mm × 25 mm × 285 mm) are removed from the molds after 24 h. The samples are placed in water (totally immersed, for 24 h, temperature 80 °C). After 24 h, the zero reading is taken and recorded. After the zero reading, the samples are relocated from water into 1N NaOH solution (totally immersed, temperature 80 °C). Subsequent measurements of the test specimens shall be performed periodically at least every 2 days for 14 days after the zero reading at approximately the same time. More often, measurements should provide more accurate results compared to the ASTM C1260 [17].

#### 2.2.3. The Evaluation of the ASR Process

The evaluation that is used in this research was based on TP137 [25] and consists of a simple equation; the calculation of which will determine the length change. The length change value of each test specimen is calculated separately and rounded to the nearest 0.001% of the length. The L value is given as the average of three bodies rounded to the nearest 0.001% of the length.

Stumbling-block in this case is a human factor. Measurements must meet strict conditions, such as a perfect temperature environment or accurate measurement and treatment. All the measurements were run for 28 days (almost two weeks longer than demanded in both standards) to ensure the most accurate and informative results. 

## 3. Results

### 3.1. Chemical Composition and Ecotoxicity

For testing the chemical and ecotoxicological properties, 27 cubes (three cubes of each mixture with the dimensions 50 mm × 50 mm × 50 mm) were made. The tested samples were also GP and reference SF, which were prepared in the laboratory (each 100 g). Leachates were prepared as follows. Table 2 shows the concentration of the selected elements that were released in leachates from the GP and HPC samples. The composition of GP leachates varied significantly. The SGP leachate showed the highest content of silicon. The GPP leachate, which contained an increased concentration of aluminum, was classified as an ecotoxic material. On the contrary, the chemical composition of HPC leachates was more consistent for most samples. In the GPP HPC 100 leachate, the highest concentration of calcium and silicon was found while the lowest amount of potassium was found. This sample also had the highest pH value (11.4). All the HPC leachates were considered environmentally safe. 

### 3.2. Alkali-silica Reaction Process

**Figure 1:**
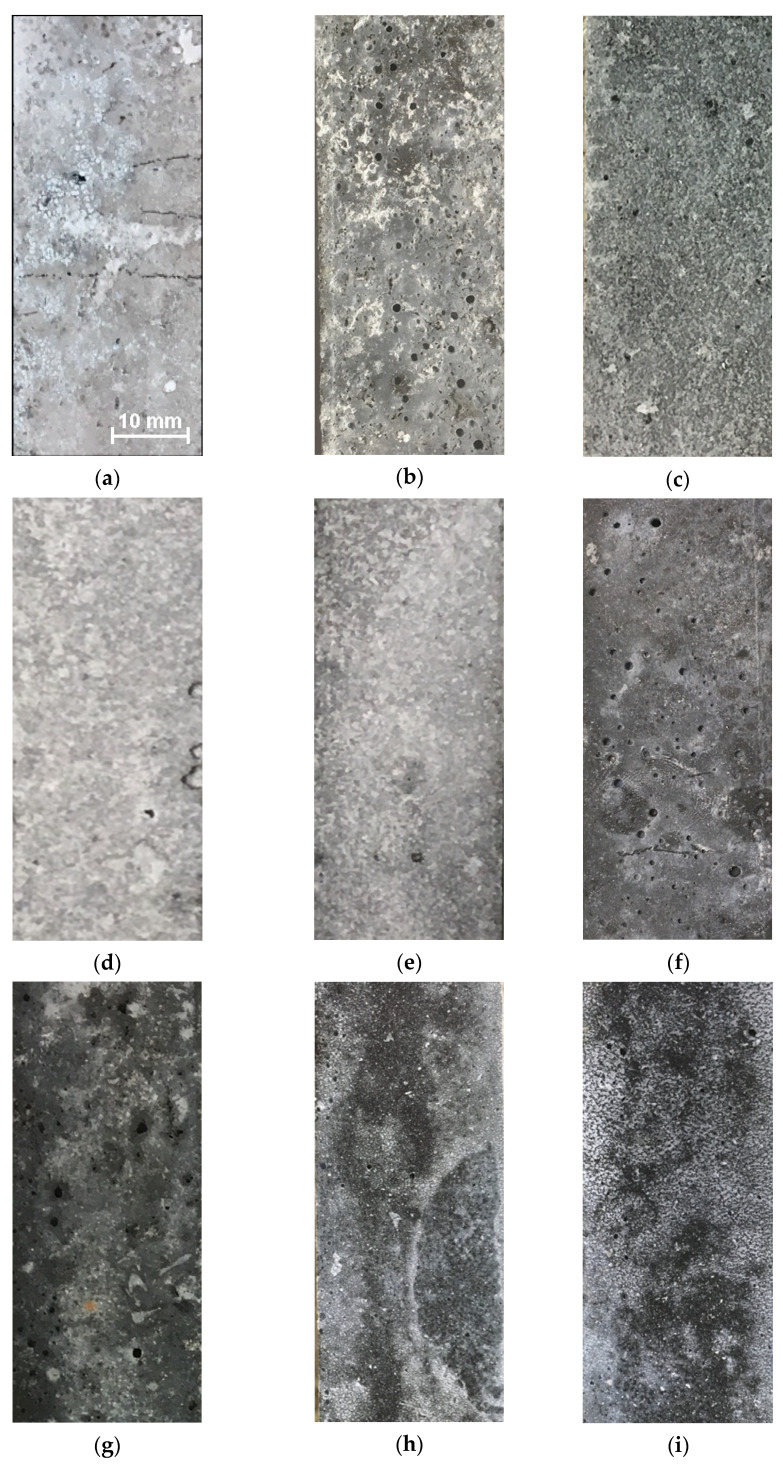
Samples surfaces after ASR testing process according to ASTM C1260 (2020/2021): (**a**) REF HPC; (**b**) GPP HPC 50%; (**c**) GPP HPC 100%; (**d**) CWG HPC 50%; (**e**) CWG HPC 100%; (**f**) SGP HPC 50%; (**g**) SGP HPC 100%; (**h**) SGJ HPC 50%; (**i**) SGJ HPC 100%.

The specimens for ASR testing were prepared according to ASTM C1260 and T137 standards. A total of nine concrete mixtures were prepared for testing, of which eight were alternative with the use of different types of powdered glass and one was used as a reference mixture. Samples of size 25 mm × 25 mm × 285 mm were subjected to tests according to two standards, always in a set of three pieces. All the samples passed the experimental process in one piece, which is evident in Figure 1. Small cracks (up to 1 cm) appeared on one reference sample (mixture REF HPC), mainly coming from the edges of the sample. No changes in the surface structure were visible in the other samples.

**Figure 2:**
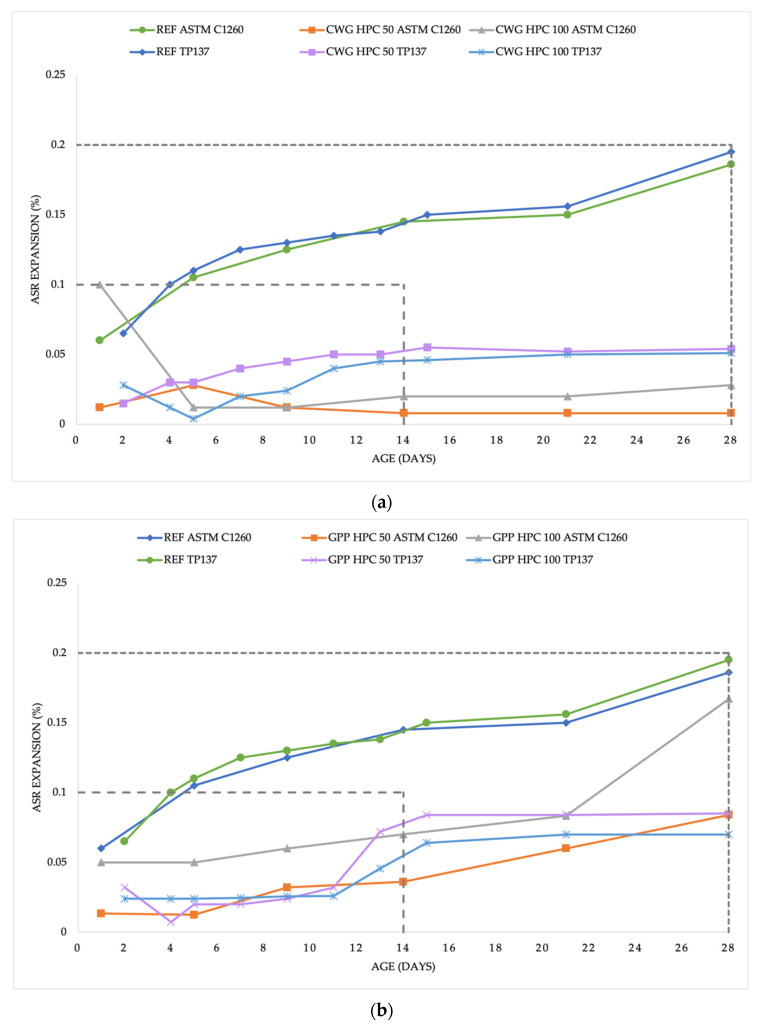

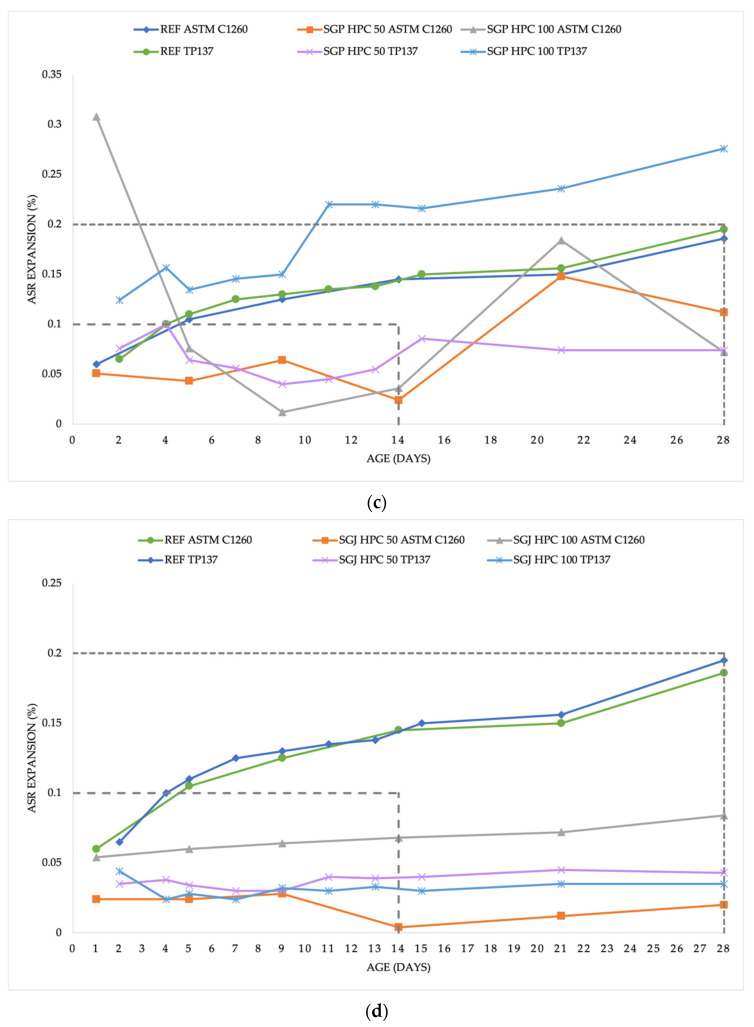
Dependence of the alkali-silica reaction process on time according to ASTMC1260 and TP137: (**a**) CWG HPC; (**b**) GPP HPC; (**c**) SGP HPC; (**d**) SGJ HPC.

The measurements were recorded in three decimal places. All the results were graphed and divided according to the standards that were used. A summary of all the results is clearly shown in Figure 2a–d. Due to the large amount of measured data, for clarity, the figures were divided according to the glass replacement that was used, that is the individual glass substitutes were compared with each other according to the tests that were performed. The 50% and 100% replacements are combined into one figure (CWG, GPP, SGP, and SGJ), and each figure also contains a reference sample for comparison (REF).

The ASTM C1260 standard specifies certain values according to which it is possible to assess whether the tested mixture is safe from the point of view of ASR, whether it is necessary to test it further, or whether it is dangerous from the point of view of ASR. In case the mixture is below the expansion value of 0.1% after 14 days, the standard indicates the harmless behavior of the concrete. After this basic period, which both standards indicate for testing, it is advisable to continue testing for at least another 14 days. If the expansion values do not exceed 0.1% after 28 days, it is still possible to declare the mixture as harmless. If the values are in the range of 0.1–0.2%, it is advisable to subject the mixture to further testing. However, if the expansion value at the age of 28 days is greater than 0.2%, the mixture may be at risk of a potential alkali-silica reaction. In this case, it would be advisable to switch to long-term precise testing of the mixture.

The results of this research vary from harmless mixtures to those with potential risk of ASR. The reference sample exceeded the safe value of 0.1% after 14 days but was still within the 0.2% zone after 28 days. From this it can be concluded that further testing is necessary, which is planned in the next phase of this research. Although the reference mixture that was used in this work has been developed for several years, the risk of an alkali-silica reaction has never been verified, and therefore, this research is also being tested as a basis for improving the reference mixture.

The CWG HPC mixtures had similar results according to both standards. All the outputs met the condition to be considered ASR innocuous materials. After 14 days, according to ASTMC1260, samples that were made from the mixture CWG HPC 50 were below 0.01%, while the values of the CWG HPC 100 mixture were around 0.02%. The results according to TP137 were measured similarly on all samples of both mixtures, around 0.05%. In all cases, after this obligation, the expansion process was stabilized, and the samples no longer showed signs of expansion.

The results with the replacement of silica flour with photovoltaic glass powder differed slightly and did not confirm 100% agreement in the results. Although both mixtures were tested according to TP137 and GPP HPC 50 was tested according to ASTM C1260 showed fairly similar results, GPP HPC 100 tested according to ASTMC1260 differed and after 21 days there was a sharp increase in the expansion instead of the expected stabilization. The increased value ended up at 0.168% and pointed out the requirement of further testing. The other outputs varied from 0.068% to 0.082% and were almost stable after 14 days. The highest increasing tendency was almost identical until the 14th day.

The most diverse course was recorded in the testing of SGP HPC-type mixtures. The expansion took place in a nonstandard and diverse manner in the three measured data. Although the samples were measured according to ASTM C1260, both recorded an unusual fluctuation at 21 days and a subsequent comparison at 28 days and TP137 recorded an almost continuous increase but in diametrically different values. While SGP HPC 50 according to TP137 stabilized after only 14 days and remained almost unchanged at 0.08% until the 28th day, SGP HPC 100 experienced a sharp increase already between the 9th and 11th day and increased dramatically above the safe zone. There was a stabilization at the value of 0.22%; however, after the 21st day, growth began to appear again and continued exponentially up to the value of 0.278%. However, no cracks were still visually evident. The mixture with a 50% replacement of quartz flour according to both standards showed that the limit of 0.1% was met after 14 days; however, due to the following outputs (between 14 and 28 days), it is advisable to consider long-term tests at the same time as repeating the ones that were already carried out.

In all respects, the SGJ HPC samples had very balanced and stable results (especially compared to those of the aforementioned SGP HPC). According to TP137, there was no noticeable fluctuation during the entire test period; both tested mixtures (50% and 100% replacement) remained around the value of 0.04% until the 28th day, when the test was completed. The results were very similar for all samples of both mixtures. The test according to ASTM C1260 had a slightly more varied result but was correlated with the results according to TP137. During the testing, SGJ HPC 50 was around 0.005–0.028% with stabilization at around 0.02%, while SGJ HPC 100 was around 0.054–0.083% and due to continued moderate growth, we cannot talk about a specific stabilization value.

## 4. Discussion

### 4.1. Chemical Analysis

From a chemical point of view, there are certain types of compounds that can affect ASR, its formation, and its overall process. In this study, it is mainly SiO_2_, however, due to the lower amount in all the GP samples (SGP, SGJ, CWG, and GPP) compared to the reference sample SF, no negative impact is expected. High proportions also appear for CaO and Na_2_O. In the case of CaO, no risk is assumed because the test takes place in an alkaline environment, and the same can be considered for Na_2_O, where the occurrence of an undesirable chemical reaction is not expected. The results are based on previous research [29] and are summarized in Table 3. 

On the other hand, the composition of a pure solid material does not necessarily indicate the resulting leachability and the internal chemical processes in HPC. Although the highest Si content was found in the reference samples (SF; REF), as Table 2 shows, the highest Si content in GP leachates was found in SGP. In HPC leachates, the Si content in SGP HPC 100 and GPP HPC 100 was higher than in the reference, REF HPC. These observations are not unexpected when the HPC samples are considered as various chemical mixtures with various porosities (Figure 1).

### 4.2. Alkali-silica Expansion

There are many different methods worldwide for testing and then evaluating the alkali-silica reaction. In the Czech Republic, the standard is valid since 1967 [19]. The tested specimens are 40 mm × 40 mm × 160 mm, and the experiment is long-term—the intermediate readings are usually done every month (for 3 months) and then every 3 months. If necessary, the experiment is continuously observed up to 18 months. The conditions are mild (humid air at 40 °C). The British standard BS 812-123 [18], which has a similar character, can be compared with the Czech standard. The British experimental design is used in research [30] and requires milder conditions (38 °C) and a longer testing period—the minimum is 52 weeks. However, measurements are taken until the values stabilize. Due to the high number of mixtures that were tested and the high time and space requirements, the short-term experiments were selected into this work.

**Figure 3:**
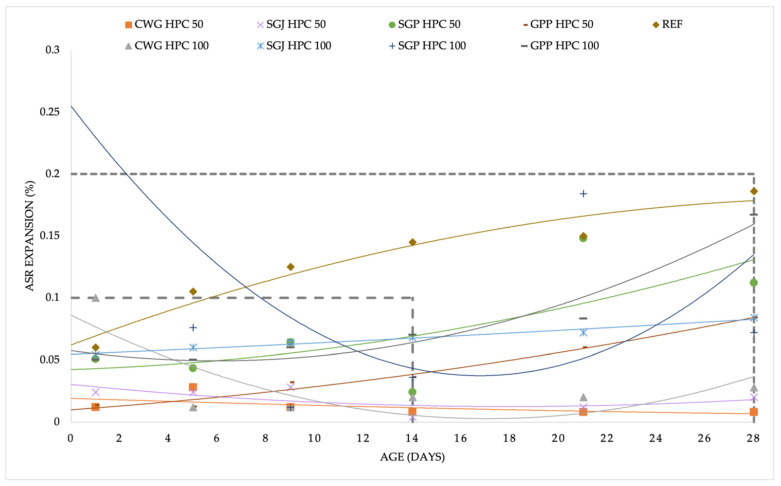
Summarized results of the dependence of the ASR expansion on time according to the ASTM C1260 standard.

**Figure 4:**
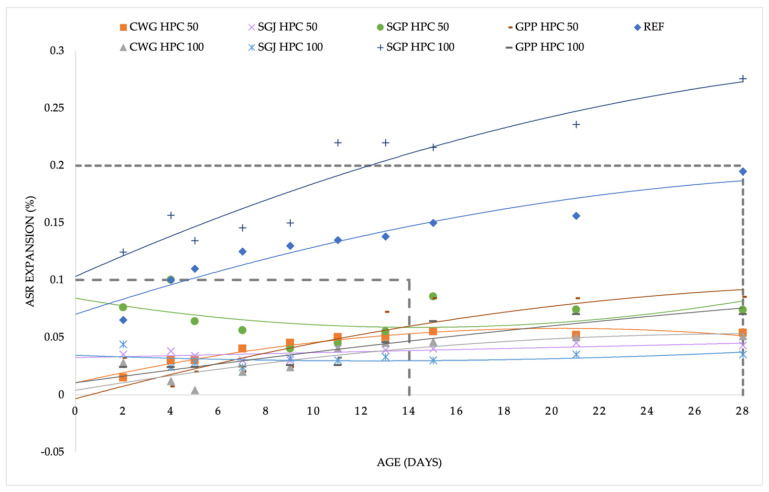
Summarized results of dependence of ASR expansion on time according to the TP137.

Nevertheless, measurement of expansion that is caused by the alkali-silica reaction is a lengthy process that does not require one result and does not end with one result. It is necessary to monitor the course of the measurements, monitor the points at which sudden changes occur, and ideally, wait until the end of the process of stabilization of the measured values. Given the unusual deflections, it is convenient to fit a curve through the resulting points to visualize the testing process; in this case, a second-degree polynomial curve was used. In Figure 3, there is a summary of the results that were established after the ASTM C1260 test. According to the standard definition of the potential risk of ASR, most of the mixtures passed the test successfully and were found to be harmless from the point of view of ASR. A similar result was obtained from the TP137 standard (Figure 4). Except for one mixture with a glass subsitute (SGP HPC 100), all the mixtures were below the critical value of 0.1% after 14 days.

**Figure 5:**
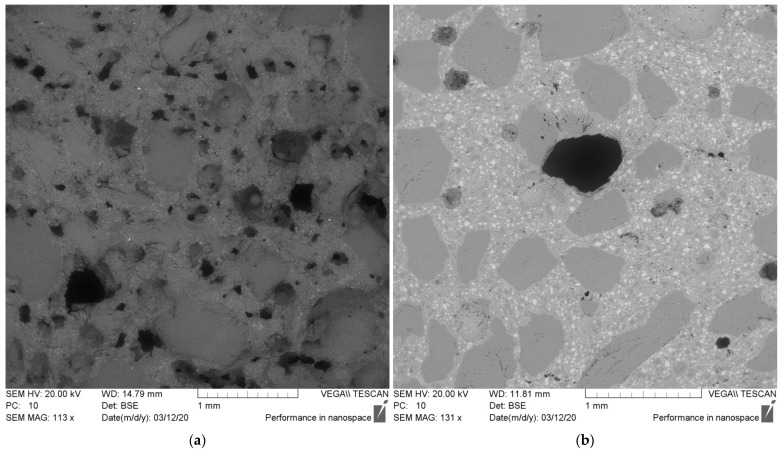
Microstructure after ASR testing. (**a**) GGP HPC 100, (**b**) REF HPC.

Relative humidity (RH) expresses the water content in structures that are affected by ASR; nevertheless, it is known that the measurement of RH in the field is often inaccurate and uncertain, and therefore, the degree of capillary saturation (DCS) is often a more appropriately used method. The relationship between these quantities is influenced by several factors, the most important of which is the water-cement ratio [22,31]. With a lower w/c concrete, the desorption isotherm is not as steep as with a higher w/c concrete, which means that the RH decreases less with higher porosity than with lower porosity [32]. During the investigation, the cross sections of the two samples were compared after the test according to ASTM C1260 (Figure 5). GPP HPC 100 is highly porous in comparison with REF HPC. It has been found that at w/c ≥ 0.45, internal moisture develops, and thus the potential development of ASR occurs. Another study is on the lower number—w/c < 0.4 [32]. The mixtures that were proposed in this work were, therefore, designed with a w/c of 0.25–0.27 according to the specification of the given mixture and the glass dust that was used. The low rate of diffusion and the lack of moisture can reduce the possible expansion of ASR with a lower w/c [22]. This premise has been confirmed in this research. 

According to Dhir et al. [20], there is no relationship between the amount of glass that is used and the expansion that is caused by ASR. At the same time, he claims that metakaolin, fly ash, or silica fume can also affect the ASR expansion. In this work, in the proposed mixtures of the type GPP HPC 50 and GPP HPC 100, microsilica was removed from the composition of the mixture. In the first pilot test, cracks formed during the setting of the concrete. The mixture was subsequently optimized, and one of the steps in the new mix was the omitting of silica fume. Therefore, this work confirms the assumption of a possible negative effect of silica fume on ASR. But, of course, it depends on the chemical properties of the material [33].

In an alkaline environment, the surface of Si-OH groups of siliceous materials ionizes, thereby acquiring a multiple negative charge. Among the most important aspects of the environment are pH values and the content of NaCl, BaCl_2_, or other neutral salts. As the concentration of these salts and pH increase, the intensity of the negative charge increases. In an alkaline environment with such a high ionic charge, hydroxyl ions OH^−^ are also released, entering deeper into the material, and penetrate the center of the material. When OH^−^ penetration slows down, the ASR rate also slows down. However, the source of ions does not have to be the only solution that is used [16]. If there are enough calcium ions in the solution, the so-called C-S-H gel is formed, which causes cracks in concrete. Changes in the pH occur when the calcium ions Ca^2+^ or SiO_2_ in the solution come into contact [34]. This advanced form of ASR was recognized for one reference sample, although the values that were measured during the experiment indicated higher length changes for the SGP HPC 100 mixture (values measured according to TP137). The reason may be precisely the higher content of Si in SF and SGP.

Based on the testing of GP samples, it is not possible to estimate in advance how they will behave as a replacement component in the HPC mixture. Therefore, it is advisable to experimentally verify this behavior repeatedly of both GP and HPC and analyze the possibility of dependence and its possible nature. 

In the aggressive conditions that are specific for both experiments that were performed (ASTM C1260 and TP137), it is possible that sudden fluctuations after the standard test period are caused by the extended expansion horizon. These were the SGP HPC 50 and SGP HPC 100 mixtures according to ASTM C1260 that experienced expansion between 14 and 28 days (Figure 3). However, according to TP137 (Figure 4), a very continuous increase was observed throughout the experiment with SGP HPC 100, and it may be an inappropriate material to be used as a substitute for SF, leading to dangerous expansion that is caused by ASR. However, the directions for further research may vary—apart from the standard option of long-term research (which was carried out by Dhir et al. [20]). In recent years, other directions have appeared, namely numerical calculations of the expansion that is caused by ASR that were presented mainly by Zhuang et al. [35,36].

## 5. Conclusions

This work compared newly designed mixes and compares international and national testing standards. In contrast to the other research works, this work deals with newly designed mixes that are based on specific types of glass. Therefore, great emphasis is placed on the chemistry and toxicity of the given materials, as it is considered one of the key factors to decipher the chemical processes that are associated with ASR.

The novelty of this research lies mainly in the innovative approach from the point of view of testing, where the testing takes place according to the latest standards, comparison between them, and at the same time, in the importance that is hidden in the use of glass materials in the Czech Republic as a local raw material.

The main contributions and findings of this work include the following points:

- The SGP leachate showed the highest content of silicon.
- The GPP leachate was classified as an ecotoxic material.
- All HPC leachates were considered environmentally safe.
- After ASTM C1260 testing, cracks appeared on one reference sample (up to 1 cm).
- No changes on the surface were observed after TP137 testing.
- GPP HPC 100 and REF HPC expansion after an extended experiment period (28 days) was between 0.1 and 0.2%. Further testing is necessary.
- SGP HPC 100 expansion after standard 14 days period was above 0.2%. Mixture may be at risk of a potential alkali-silica reaction.
- According to the standards, the conditions to exclude the occurrence of an ASR in the mixtures CWG HPC 50, CWH HPC 100, SGJ HPC 50, SGJ HPC 100, SGP HPC 50, and GPP HPC 50 were fulfilled.

In general, it is necessary to be careful with the assumption that the replacement of fine particles is more appropriate than the replacement of coarse aggregate in concrete. Although the results of this research point to the confirmation of this premise, it can only be an extension of the expansion time horizon. However, this consideration needs to be verified by long-term testing or/and numerical methods.

## Figures and Tables

**Table 1:** Composition of concrete mixtures.

Content [kg]	REF HPC	CWG HPC 50	CWG HPC 100	SGJ HPC 50	SGJ HPC 100	SGP HPC 50	SGP HPC 100	GPP HPC 50	GPP HPC 100
Cement I 42.5 R	680	680	680	680	680	680	680	650	650
Fine sand 1/6	576	576	576	576	576	576	576	600	600
Coarse sand 6/12	384	384	384	384	384	384	384	600	600
Microsilica	175	175	175	175	175	175	175	-	-
SF	325	162.5	-	162.5	-	162.5	-	120	-
CWG	-	162.5	325	-	-	-	-	-	-
SGJ	-	-	-	162.5	325	-	-	-	-
SGP	-	-	-	-	-	162.5	325	-	-
GPP	-	-	-	-	-	-	-	120	240
Plastificators	29	29	29	29	29	29	29	30	30
Water	171	171	171	171	171	171	171	180	180

**Table 2:** Chemical and ecotoxicological properties of GP and HPC leachates. S—safe, E—ecotoxic (10% leachate caused ≥ 50% effect in at least one ecotoxicity test—water flea, algae, duckweed).

Element [mg/L]	SF	CWG	SGJ	SGP	GPP	REF HPC	CWG HPC 100	SGJ HPC 100	SGP HPC 100	GPP HPC 100
Si	24.0	14.8	8.2	48.7	14.7	3.0	2.5	2.3	3.6	5.2
Na	0.7	128.3	194.7	50.6	87.1	2.2	3.0	6.2	1.2	3.6
K	1.9	4.5	110.5	32.7	0.7	18.6	15.6	10.8	18.7	8.8
Ca	3.4	5.7	3.0	1.5	6.1	20.6	26.5	20.4	20.3	54.2
Al	˂0.8	3.0	7.9	˂0.8	27.9	˂0.8	˂0.8	˂0.8	˂0.8	˂0.8
pH	6.8	10.5	10.9	11.1	10.3	11.0	11.1	11.0	10.9	11.4
Ecotoxicity	S	S	S	S	E	S	S	S	S	S

**Table 3:** Chemical analysis of glass powder on the basis of X-ray fluorescence. All values are expressed as %.

Type	Material	SiO_2_	Al_2_O_3_	Na_2_O	CaO	Fe_2_O_3_	K_2_O	Sum
Reference	SF	99.68	0.17	<0.005	0.03	0.03	<0.005	99.9
Glass	CWG	68.94	2.06	14.50	10.50	0.37	0.75	97.1
	SGJ	65.68	1.38	12.27	5.83	0.15	8.60	93.9
	SGP	63.58	0.51	16.61	2.64	0.08	7.02	90.4
	GPP	60.35	19.89	10.36	5.47	0.77	<0.005	96.8

## Data Availability

The data are available in a publicly accessible repository.
